# Whole-Exome Sequencing Identifies Novel GATA5/6 Variants in Right-Sided Congenital Heart Defects

**DOI:** 10.3390/ijms26052115

**Published:** 2025-02-27

**Authors:** Gloria K. E. Zodanu, John H. Hwang, Jordan Mudery, Carlos Sisniega, Xuedong Kang, Lee-Kai Wang, Alexander Barsegian, Reshma M. Biniwale, Ming-Sing Si, Nancy J. Halnon, UCLA Congenital Heart Defects-BioCore Faculty, Wayne W. Grody, Gary M. Satou, Glen S. Van Arsdell, Stanly F. Nelson, Marlin Touma

**Affiliations:** 1 Neonatal Congenital Heart Laboratory, Department of Pediatrics, David Geffen School of Medicine, University of California, Los Angeles, CA 90095, USA; kafsel05@gmail.com (G.K.E.Z.); johnhhwang92@gmail.com (J.H.H.); xkang@mednet.ucla.edu (X.K.); barsegian88@gmail.com (A.B.); 2 Department of Pediatrics, David Geffen School of Medicine, University of California, Los Angeles, CA 90095, USA; rbiniwale@mednet.ucla.edu (R.M.B.); nhalnon@mednet.ucla.edu (N.J.H.); wgrody@mednet.ucla.edu (W.W.G.); gsatou@mednet.ucla.edu (G.M.S.);; 3 Department of Human Genetics, David Geffen School of Medicine, University of California, Los Angeles, CA 90095, USA; 4 Department of Surgery, David Geffen School of Medicine, University of California, Los Angeles, CA 90095, USA; msi@mednet.ucla.edu; 5 Department of Pathology and Laboratory Medicine, David Geffen School of Medicine, University of California, Los Angeles, CA 90095, USA; 6 Molecular Biology Institute, University of California, Los Angeles, CA 90095, USA; 7 Children’s Discovery and Innovation Institute, University of California, Los Angeles, CA 90095, USA; 8 Eli and Edyth Broad Stem Cell Research Center, University of California, Los Angeles, CA 90095, USA; 9 Cardiovascular Research Laboratories, David Geffen School of Medicine, University of California, Los Angeles, CA 90095, USA

**Keywords:** GATA, transcription factor, zinc finger-containing protein, pulmonary valve stenosis, right-sided CHD, right ventricle hypoplasia, whole-exome sequencing

## Abstract

One out of every hundred live births present with congenital heart abnormalities caused by the aberrant development of the embryonic cardiovascular system. The conserved zinc finger transcription factor proteins, which include GATA binding protein 5 (GATA5) and GATA binding protein (GATA6) play important roles in embryonic development and their inactivation may result in congenital heart defects (CHDs). In this study, we performed genotypic–phenotypic analyses in two families affected by right-sided CHD diagnosed by echocardiography imaging. Proband A presented with pulmonary valve stenosis, and proband B presented with complex CHD involving the right heart structures. For variant detection, we employed whole-genome single-nucleotide polymorphism (SNP) microarray and family-based whole-exome sequencing (WES) studies. Proband A is a full-term infant who was admitted to the neonatal intensive care unit (NICU) at five days of life for pulmonary valve stenosis (PVS). Genomic studies revealed a normal SNP microarray; however, quad WES analysis identified a novel heterozygous [Chr20:g.61041597C>G (p.Arg237Pro)] variant in the *GATA5* gene. Further analysis confirmed that the novel variant was inherited from the mother but was absent in the father and the maternal uncle with a history of heart murmur. Proband B was born prematurely at 35 weeks gestation with a prenatally diagnosed complex CHD. A postnatal evaluation revealed right-sided heart defects including pulmonary atresia with intact ventricular septum (PA/IVS), right ventricular hypoplasia, tricuspid valve hypoplasia, hypoplastic main and bilateral branch pulmonary arteries, and possible coronary sinusoids. Cardiac catheterization yielded anatomy and hemodynamics unfavorable to repair. Hence, heart transplantation was indicated. Upon genomic testing, a normal SNP microarray was observed, while trio WES analysis identified a novel heterozygous [Chr18:c.1757C>T (p.Pro586Leu)] variant in the *GATA6* gene. This variant was inherited from the father, who carries a clinical diagnosis of tetralogy of Fallot. These findings provide new insights into novel *GATA5/6* variants, elaborate on the genotypic and phenotypic association, and highlight the critical role of GATA5 and GATA6 transcription factors in a wide spectrum of right-sided CHDs.

## 1. Introduction

The first organ to form during embryogenesis is the heart. This occurs through a complex process involving an intricate network of transcriptional regulators that orchestrate several molecular processes [1,2]. Congenital heart defects (CHDs) describe the structural and functional abnormalities that occur during the embryonic stage of cardiac development, and they are the most frequently occurring birth defects, affecting 1 in every 100 live births [3,4]. Even though the exact causes of most CHDs remain unknown, genetic and environmental factors and their interactions have been implicated as the main causes of CHD [5]. There have been remarkable efforts to elucidate the mechanisms involved in normal cardiac development and malformation to better understand the formation of these heart defects [5]. Literature evidence has linked some heart defects with pathogenic variants in genes that encode the zinc finger (ZnF)-containing GATA binding protein (GATA) family of transcription factors [6,7].

### 1.1. Molecular Structure of the ZnF-Containing GATA Transcription Factors

There are approximately 2600 genes in the human genome that encode transcription factor proteins that possess deoxyribonucleic acid (DNA)-binding ability [8]. These transcription factors are distinguished by the structural differences in their DNA-binding domains [9]. ZnF proteins are small proteins that have zinc molecules in their structure that serve to coordinate the assembly of the protein and maintain the stability of the domains [10]. ZnF transcription factors have finger-like sites that help recognize and directly bind to the DNA sequences of target genes [11]. They perform a wide range of biological functions including transcriptional regulation, DNA replication and repair, metabolism, cell proliferation, and apoptosis [10].

**Figure 1:**
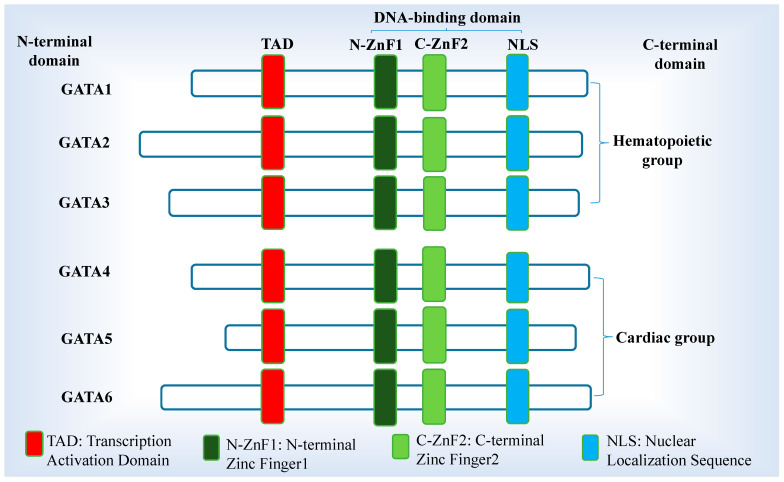
Schematic diagram of structural domains of the GATA transcription factors. GATA 1-6 (subdivided into hematopoietic and cardiac groups) comprises a conserved DNA-binding domain consisting of two similar zinc fingers (N-ZnF1, C-ZnF2). The two distinct ZnFs and the nuclear localization sequence (NLS) collectively form the DNA-binding domain. The N-terminal consists of the transcription activation domains (TADs). The C-terminal acts as the DNA-binding site and functions as a domain for nuclear localization.

The GATA family transcription factors, which were originally discovered due to their ability to recognize the consensus DNA sequence, (A/T)GATA(A/G), play vital roles during development [1]. These factors are particularly dependent on two conserved ZnFs with their flanking sequences, and their perturbations may lead to several forms of congenital abnormalities, including CHDs [4,12,13]. The GATA family consists of six members (GATA1-6) that have the potential to bind to the consensus DNA-binding sequence (A/T)GATA(A/G) in the enhancer and promoter regions of their target genes. This sequence is highly conserved, and its binding is mediated by two similar ZnF domains (ZnF1&2) that are common to all the GATA groups [1,2,14,15,16] (Figure 1). Each of these ZnFs has a distinct function and binds to target sites independently. Because of their proximity to the amino or carboxyl terminus, the two connecting and nearby ZnFs are labeled as C-terminal and N-terminal zinc fingers (C-ZnF2, N-ZnF1) [14,17,18]. While the C-ZnF2 is essential for the identification and the binding of the GATA consensus sequence with high affinity, the N-ZnF1 either binds the GATA recognition sequence or stabilizes their interactions with other diverse proteins [14,19,20,21] (Figure 1). The amino acid sequences of the N-terminal and C-terminal domains have less similarity, despite the ZnF domains being highly conserved (>70%) [21]. Additionally, found at the C-terminus is the nuclear localization sequence (NLS), and at the N-terminus is the transcription activation domain (TAD). These domains are necessary for appropriate nuclear localization and transcriptional activation [14,17,18].

### 1.2. Biological Roles of GATA Proteins

Different cell types depend on GATA 1-6 proteins for differentiation, proliferation, and survival [18,22,23]. Depending on their location, composition, and biological purpose, they are divided into two sub-groups [18,22]. GATA1/2/3 is mainly expressed in developing blood cells and works by altering the expression of genes relevant to differentiation in hematopoiesis. They also aid in the basic development of the organs and are also present in the brain, spinal cord, and inner ear [18,24,25,26]. As shown below, the discovery of the loss of function mutations in *GATA1/2/3* has revealed evidence of several clinical diseases [27]. An interesting aspect of the hematopoietic factor genes is the GATA1 which encodes a transcription factor required for the healthy development of blood cells and is connected to the onset of Down syndrome [28]. A percentage of 40 to 63.5% of people with Down syndrome (DS) have CHD. Even though there is currently no proof linking GATA1 mutations to CHDs in DS, CHD is a major cause of death and morbidity rate among those affected with DS [29,30,31] (Table 1).

GATA4/5/6 are essential in regulating tissue-specific gene expression mainly in the cardiovascular system. They also present in mesodermal and endodermal organs and contribute to the formation of liver, pancreas, lung, gonad, and gut [18,24,25,26]. Despite the cardiac group of GATA transcription factors being expressed in an overlapping manner within similar anatomical domains, they exhibit discrete spatiotemporal patterns throughout embryogenesis [23]. Even in adult cardiac myocytes, GATA4 and GATA6 remain expressed, in contrast to GATA5 [7]. As a result, their genetic mutations are associated with diverse CHD phenotypes [8]. However, due to the complexity of its functional mechanism and crucial roles during late cardiac development, including septation and valve formation, GATA4 has been the subject of the majority of investigations [32]. In addition to cardiac defects, *GATA4* mutation causes congenital hernias, monogenic diabetes, and abnormal testicular development [27]. The different abnormalities that resulted from the deactivation of their encoding genes in mice highlighted the unique involvement of GATA4/5/6 in various stages of heart development [33,34,35]. Indeed, the maturing cardiac system is highly responsive to the levels of GATA4/5/6, implying that these transcription factor genes have a point at which they can collectively work together to control downstream cardiac genes that are necessary for normal heart formation [20,36]. Jointly, the GATA4/5/6 proteins may bind similar nucleotide sequences in genomic DNA and control similar target genes [15,20,37,38]. Using data from PubMed search and Google Scholar reviews, Whitcomb et al. (2020) [26] gathered cardiac abnormalities linked to human *GATA4/5/6* mutations (Table 2).

Herein, we performed genotypic–phenotypic analyses in two probands affected with novel *GATA5* and *GATA6* variants and congenital heart abnormalities, mostly affecting the right cardiac structures.

## 2. Results

### 2.1. History and Clinical Course

#### 2.1.1. Case A

**Figure 2:**
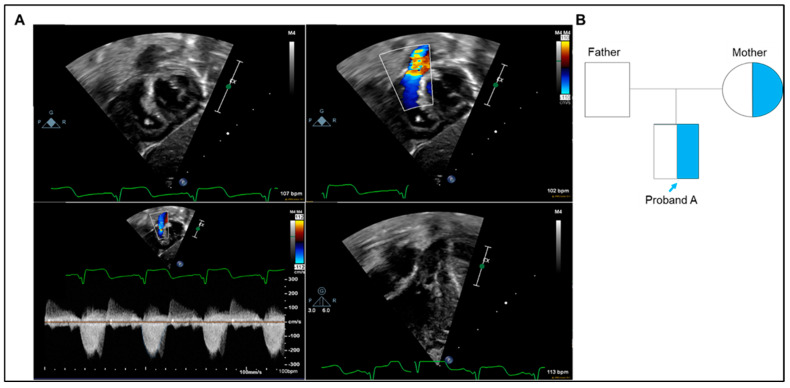
Clinical data of proband A. (**A**) Neonatal echocardiogram findings. Top left: Subcostal short-axis 2D echocardiography for the RVOT in systole showing crowding at the level of the pulmonary valve. Top Right: Color Doppler interrogation of the RVOT showing turbulence at the level of the pulmonary valve. Bottom left: Spectral Doppler display of a continuous-wave sample of the RVOT showing fixed mild obstruction and regurgitation. Bottom right: Anteriorly displaced apical four-chamber view showing thickened pulmonary valve with possible tethering of the left-sided leaflet. (**B**) Family pedigree of proband A. The proband and his mother are heterozygous for the *GATA5* variant.

Proband A is a full-term male infant who was born via spontaneous vaginal delivery to healthy non-consanguineous parents. His delivery and early postnatal course were notable for his stunned appearance and delayed initial cry that responded quickly to routine resuscitation. However, he failed the routine congenital heart screening test, mandating admission to the neonatal intensive care unit (NICU) at five days of life. Upon admission, a systolic murmur was present, and echocardiogram imaging showed pulmonary valve stenosis (PVS) with domed, thickened valve leaflets, a patent foramen ovale with bidirectional shunting, and a small patent ductus arteriosus with right to left shunting peak at 34 mmHg (Figure 2A). No dysmorphic features or other malformations were identified besides the PS. Although the PVS was severe, adequate pulmonary blood flow was maintained with normal oxygen saturation levels on room air, and therefore, the infant did not require prostaglandin E2 (PGE2) infusion. However, he later developed supra-systemic right ventricular (RV) pressure with right ventricular outflow tract (RVOT) obstruction that prompted cardiac catheterization and pulmonary valvuloplasty. During the cardiac catheterization, he was noted to have elevated ST segments on electrocardiogram (EKG) monitoring that was thought to be related to right heart strain. Following the procedure, troponin was found to be elevated with ongoing ST segment changes. Subsequent serial echocardiograms revealed improved RV filling and function without pericardial effusion. Follow-up troponin levels trended to normal, and serial EKGs showed gradual resolution of ST segment changes.

There was no family history of CHD. However, the mother had a remote history of smoking cigarettes in the past but quit 8 years ago. Her father and brother were diagnosed with “innocent murmurs”. The family history was unremarkable otherwise (Figure 2B).

#### 2.1.2. Case B

Proband B is a male premature infant born at 35 weeks gestation via spontaneous vaginal delivery who was admitted to the NICU for a prenatal diagnosis of complex CHD involving the right-sided structures. The infant emerged vigorously and did well until 5 min of life when oxygen saturation levels dropped to ~40% requiring supplemental oxygen. Subsequently, he was admitted to the NICU for close monitoring and management including immediate PGE2 infusion for ductal-dependent pulmonary blood flow achieving average oxygen saturation levels >75%.

**Figure 3:**
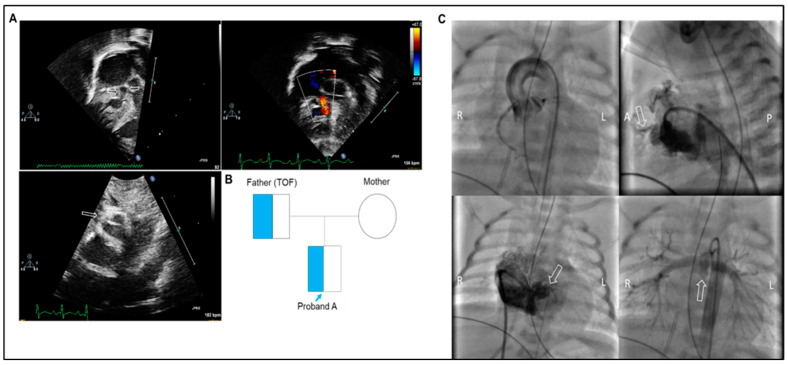
Clinical data of proband B. (**A**) Neonatal echocardiogram findings. Top left: Off-axis apical four-chamber view of the RV showing dilated right atrium and a hypoplastic RV with a hypoplastic Tricuspid valve (TV) (arrows). Top right: Color Doppler interrogation of the TV showing narrow inflow. Bottom left: Off-axis parasternal short-axis view of the RVOT showing plate-like atresia of the pulmonary valve (arrow). (**B**) Family pedigree of proband B. The proband inherited the *GATA6* variant from the affected father. (**C**) Angiocardiographic images from cardiac catheterization. Top left: Anteroposterior projection of an aortic root injection showing poor filling of the distal right coronary artery and all of the left coronary system. Top right: Lateral projection of right ventriculogram showing hypoplastic unipartite RV consisting of the inlet portion with contrast traversing anteriorly through the myocardium into the territory of the left anterior descending artery (arrow). There is no evidence of an RVOT. Bottom left: Anterior projection of a right ventriculogram showing no evidence of RVOT with hypoplastic unipartite RV with contrast traversing leftward through the myocardium into the territory of the left anterior descending artery (arrow). Bottom Right: Anterior projection of an aortic arch injection showing adequate size pulmonary artery and branch pulmonary arteries (arrow).

Upon admission, a postnatal echocardiogram confirmed several right-sided heart defects including pulmonary atresia with intact ventricular septum (PA/IVS), severe RV hypoplasia, tricuspid valve hypoplasia, hypoplastic right and left pulmonary arteries, along with a possible coronary sinusoid. No dysmorphic features or other malformations were identified besides the heart defects. The infant’s course in the NICU was also remarkable for respiratory insufficiency requiring intubation and assisted mechanical ventilation, pulmonary edema requiring treatment with diuretics, and pericardial effusion requiring drainage. Cardiac catheterization yielded anatomy unfavorable to repair due to an RV-dependent coronary circulation (Figure 3C). A palliative ductal stent was placed, which served to stabilize the hemodynamic status while awaiting heart transplantation. The parents have no consanguinity. Of note, the proband’s father reported a history of repaired tetralogy of Fallot (TOF) with no other family history of CHDs.

### 2.2. Genetic Testing Results

#### 2.2.1. Proband A

SNP microarray observed no copy number gains or losses of genomic regions of known clinical significance or long contiguous stretches of homozygosity.

##### Quad Whole-Exome Sequencing (WES)

Quad WES was performed using the gDNA of the proband, both healthy parents and a maternal uncle who reported a remote history of an innocent heart murmur. Exome variant analysis was performed following ACMG recommendations. The data was consistent with an XY individual. No established clinically significant variants were identified to explain our patient’s primary clinical concerns. However, a novel heterozygous [Chr20:g.61041597C>G (p.Arg237Pro)] variant in the *GATA5* gene was identified and was also present in the proband’s mother. Specifically, at transcript NM_080473.4: (c.710G>C), which is on the negative strand, position 710, the reference sequence C is replaced with G in our proband. In the corresponding protein, the arginine is substituted for a proline residue at position 237 (p.Arg237Pro) (Table 3).

Pathogenic *GATA5* variations are known to be associated with autosomal dominant and autosomal recessive CHDs [OMIM: 617912]. The heterozygous [Chr:g.61041597C>G (p.Arg237Pro)] variant in *GATA5* has not been previously observed in the general population in the ExAC database. Importantly, six out of six functional prediction algorithms (SIFT, Polyphen2 HVAR, Mutation Taster, MutationAssesor, FATHMM, and FATHMM MKL Coding) predict this variant to be damaging. As the variant call quality was high, Sanger sequencing was not required to confirm the variant. Of note, there were no regions of homozygosity (>5 Mb) identified in proband’s genome. In addition, the maternal uncle’s sample did not carry this variant.

#### 2.2.2. Proband B

##### Whole-Genome SNP Microarray

Normal chromosomal microarrays were observed with no copy number gains or losses of genomic regions of known clinical significance.

##### Trio WES

Trio WES was performed using the gDNA of the proband and both parents. Exome variant analysis was performed following ACMG recommendations. The data was consistent with an XY individual. No established clinically significant variants were identified to explain our patient’s primary clinical concerns. However, a novel heterozygous [Chr18:c.1757C>T (p.Pro586Leu)] variant in the GATA6 was identified. This variant substitutes the proline for a leucine residue at position 586 (p.Pro586Leu) in the GATA6 protein of the proband, which was found to be inherited from the affected father suggesting an autosomal dominant trait (Table 4).

Pathogenic *GATA6* variations are known to be associated with autosomal dominant CHDs [OMIM: 601656]. The variant query using a literature search in public databases indicated one report of this novel variant and was classified as a variant of uncertain clinical significance (VUS). Three out of six functional prediction algorithms predict this variant to be damaging. As the variant call quality was high, Sanger sequencing was not required to confirm the variant.

## 3. Methods

### 3.1. Human Studies

All human studies were conducted following the regulations of the University of California Los Angeles (UCLA) Institutional Review Board (IRB). Subjects or their legal representatives provided written informed consent to participate in this study. Electronic medical records, family history, and specimen collection were acquired through the UCLA Congenital Heart Defect (CHD) BioCore following the UCLA-IRB-approved protocols. Prenatal diagnoses were determined based on serial fetal sonography and echocardiography findings. Clinical diagnoses were determined based on clinical features, echocardiography imaging, and cardiac catheterization findings.

### 3.2. Whole-Genome Single-Nucleotide Polymorphism (SNP) Microarray

Whole-genome SNP microarray and oligonucleotide array were performed using genomic DNA (gDNA) isolated from peripheral blood monocytes at the UCLA Clinical Genomics Center following clinically validated CLIA (Clinical Laboratory Improvement Amendments)- and CAP (College of American Pathologists)-validated protocols. The SNP oligonucleotide array was used to assess copy number variations (CNVs), insertions, deletions, duplications, and genomic imbalances as well as neutral alterations (regions of homozygosity) that indicate an absence or loss of heterozygosity in the sample tested. The assay compared the patient’s DNA to an internal reference and an external reference from 380 normal controls using the Affymetrix Genome-Wide SNP Array CytoScan™ HD (ThermoFisher Scientific, Waltham, MA, USA) for both normalization and comparative analysis. This oligonucleotide array platform contains 2.6 million markers for CNV detection, of which 750,000 are genotype SNPs and 1.9 million are non-polymorphic probes for whole-genome coverage. The analysis was performed using the Affymetrix Chromosome Analysis Suite (ChAS) software, version 3.30.139 (r10838).

### 3.3. Whole-Exome Sequencing (WES)

The genomic DNA was extracted from peripheral blood monocytes (PBMCs) at the UCLA Congenital Heart Defects BioCore by using standard methods (Purelink Genomic DNA Mini Kit, Invitrogen, Waltham, MA, USA). Library preparation, sequencing, and data analysis were performed at the CCRD (California Center for Rare Disease) and the UCLA Clinical Genomics Center, using CLIA- and CAP-validated protocols. Genomic DNA (3 µg) samples from the proband and parents were subjected to library preparation and exome capture following the Agilent Sure Select Human All Exon 50 Mb (Agilent Technologies, Santa Clara, CA, USA) Illumina Paired-End Sequencing Library Prep Protocol. Sequencing was performed on an Illumina HiSeq4000 (Illumina, San Diego, CA, USA) as a 50 bp paired-end run. For each sample, approximately 200 million independent paired reads were generated for an average coverage of 140×± of RefSeq protein-coding exons and flanking introns (±2 bp), with at least 95% of these bases covered at ≥10×. hg19/b37 genome release by using the Novoalign function. PCR duplicates were marked using Picard. The Genome Analysis Toolkit (GATK) [39] was used for insertions and deletion (INDEL) realignment and base quality recalibration. Both single-nucleotide variants (SNVs)and small INDELs were called using a GATK-unified genotyper. All variants were annotated using the customized variant effect predictor (VEP) engine from Ensembl. Regions of homozygosity by descent were determined using PLINK, an open-source C/C++ whole-genome association studies (WGASs) tool set. Rare variants with a minor allele frequency of <1% in public databases were retained for further analysis [40].

### 3.4. Variant Analysis

Candidate rare variants were classified based on their zygosity and pattern of inheritance, location within the gene, conservation scores, population and allele frequencies (ClinVar), predicted consequence at the protein level and structural domains, pathogenicity prediction in silico tools, evidence from functional studies and animal models, and disease spectrum, following the American College of Medical Genetics (ACMG) and the Association for Molecular Pathology (AMP) Guidelines for Interpretation of Sequence Variants [41]. All variants were interpreted in the context of the patient’s phenotype and further prioritized, but not restricted to, a primary gene list which was based on keywords including CHDs and generated using the UCLA Cardiac Disorders Gene Panel (Jan 2015), the HGMD Gene Mutation Database Professional Version 2019.1, and OMIM (Online Mendelian Inheritance In Man). Variants were dismissed if they were predicted to be tolerant, i.e., have a low impact on protein structure or function, or have been reported in the GnomAD database. Six functional prediction algorithms were used to predict the damaging effect of a given variant: SIFT, Polyphen2 HVAR, Mutation Taster, Mutation Assessor, FATHMM, and FATHMM MKL Coding. Finally, the technical quality of the candidate variants was confirmed using the Integrative Genomics Viewer (IGV) v2.16.0 [42].

## 4. Discussion

### 4.1. GATA5 Variant and Pulmonary Stenosis in Proband A

On admission, proband A was diagnosed with severe congenital pulmonary valvar stenosis. A novel heterozygous[Chr20:g.61041597C>G (pArg237Pro)] variant in the *GATA5* was identified that was inherited from the mother but was absent in the father and maternal uncle.

**Figure 4:**
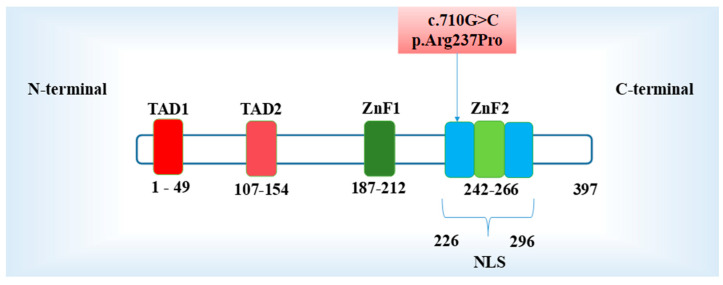
Schematic diagram of structural domains and the location of the GATA5 variant. The N-terminal consists of the two transcription activation domains (TAD1&2). A DNA-binding domain consisting of two similar zinc fingers (N-ZnF1, C-ZnF2) and the nuclear localization sequence (NLS). Upper light red box: Illustration of the GATA5 variant of interest in this study.

*GATA5* is located on chromosome 20q13.33 and encodes a protein (GATA5) composed of 397 amino acids [34]. The functional structure of GATA5 is predicted to have two transcriptional activation domains (TAD1, amino acids 1–49 and TAD2, amino acids 107–154), two adjacent ZnF domains (ZnF1, amino acids 187–212, and ZnF2 amino acids 242–266) and one nuclear localization sequence (NLS, amino acids 226–296) adjacent to the ZnF2. The two TADs are necessary for the transcriptional activity of GATA5 [34], while the NLS/ZnF2 domain serves to facilitate the nuclear localization and sequence binding (Figure 4). The [Chr20:g.61041597C>G (pArg237Pro)] variant is located in exon 4 and within the ZnF2/NLS protein domain (Figure 4). This missense variant substitutes arginine with a proline residue, potentially altering the biochemical property of the GATA5 protein. Importantly, the arginine residue makes a protein hydrophilic due to its positively charged side chain, which easily forms hydrogen bonds with water molecules. As a result, it usually appears on the surface of a protein that is exposed to the aqueous environment. In contrast, the proline residue increases the protein’s hydrophobicity, which implies that it typically resides inside the protein structure and tends to avoid aqueous environments due to its non-polar side chain. The resulting conformational changes adjacent to the ZnF2/NLS domain may potentially affect the binding activity or the nuclear translocation. Consistently, six out of six functional prediction algorithms predicted the p.Arg237Pro to be damaging, suggesting that our patient’s phenotype could be attributed to the mutant GATA5^p.Arg237Pro^ protein.

GATA5 is expressed in the myocardium, endocardium, and derived endocardial cushions of the human heart. When GATA5 is inactivated in endocardial cells during development, it results in hypoplastic hearts and partially penetrant bicuspid aortic valve formation [14,22]. According to several functional analyses, most loss-of-function variants cause *GATA5* transcriptional activity to decrease during cardiac morphogenesis, which in turn contributes to CHD [43,44]. However, despite several reports that pathogenic *GATA5* variants mostly cause CHDs (Table 5), the mechanism via which the mutant GATA5 proteins cause heart abnormalities remain poorly understood. Apart from several CHDs, loss-of-function mutations in GATA4 and GAT5 contribute to neonatal dilated cardiomyopathy that can lead to congestive heart failure and sudden cardiac failure, which is the primary reason for transplants in affected individuals [45,46].

The cardiac GATA transcription factors are expressed at several stages of heart development and are inter-regulated modularly by several cofactors to increase gene expression [32]. Therefore, the expression and the role of GATA5 partially overlap that of GATA4 and GATA6 during development, making it an important molecular factor for different congenital heart diseases [32,34]. For instance, GATA5 can integrate with other GATA factors to control cardiac gene expression [43,47,48]. *GATA5* regulation is susceptible to copy number changes in early heart development and acts before *GATA4* to control the growth of cardiac precursors [22,43]. Furthermore, the expression of the cardiac troponin T is regulated by the *GATA5*, which significantly accelerates the differentiation of mouse embryonic stem cells into cardiomyocytes [49]. It has been shown that *GATA5* expression is regulated spatiotemporally with a brief expression in early myocardial cell differentiation, endocardial cell differentiation, cushion differentiation, and valvular formation, in contrast to *GATA4* and *GATA6* that maintain expression in mature cardiomyocytes and various adult heart parts postnatally [22,35,47]. Additionally, functional dysregulation of GATA5 could result in the development of hypertension, arrhythmia, and other human diseases through different signaling pathways [50].

*GATA5* was not initially recorded on the CHD map until Laforest et al. (2011) reported the outcomes of *GATA5* deletion in mice resulting in defective valve morphogenesis and various heart defects [22,35]. Subsequently, genetic variations in human *GATA5* were associated with several CHDs, including aortic stenosis, pulmonary stenosis, double outlet right ventricle, tetralogy of Fallot (TOF), bicuspid aortic valve, ventricular septal defect (VSD), and atrial septal defect (ASD) [32,34]. As of 2016, twelve papers had been published demonstrating the connections between various *GATA5* mutations and various forms of CHDs [35,44]. However, it was difficult to ascertain the inheritance of these heterozygous variants since, in the majority of these cases, the parents’ genotypes were not examined [35,44]. More than twenty missense variants have since been identified to be linked with diverse forms of CHDs [19,35,44,45,47,48,51,52] (Table 2 and Table 5). Furthermore, there is an increased risk of CHD recurrence within families and among affected relatives [22]. Affected individuals usually present with diverse cardiac phenotypes with varying expressivity and penetrance suggesting that modifying genetic and epigenetic factors may impact the phenotypes [22].

**Table 5:** Variants of GATA5 documented in the literature.

Nucleotide Change	Protein Change	Phenotype	Mode of Inheritance	Consequence	Reference
c.710G>C g.61041597C>G novel	p.Arg237Pro	Pulmonary valve stenosis, status post pulmonary valvuloplasty, patent foramen ovale with a right to left shunting	Heterozygous	Damaging	Current paper
c.T>C missense	p. T289A novel	Atrioventricular canal defect	Heterozygous Autosomal dominant	Possibly damaging	Kassab et al., 2016 [35]
c.A>G missense	p.L233P	Coarctation of the aorta	-	Possibly damaging	Kassab et al., 2016 [35]
c.C>T missense	p.G166S	Single ventricle Tetralogy of Fallet	-	Benign	Kassab et al., 2016 [35]
c.A>G missense	p.Y142H	Double outlet right ventricle, small ventricular septal defect, mild pulmonary stenosis	Heterozygous	Possibly damaging	Kassab et al., 2016 [35]
c. T>G missense	p.T67P	Atrial septal defect, coarctation of the aorta, ventricular septal defect, patent ductus arteriosus, atrioventricular canal defect	Heterozygous	Benign	Kassab et al., 2016 [35]
g.61050383G>C missense	p.G63A novel	Pulmonary stenosis	Heterozygous	Benign	Kassab et al., 2016 [35]
c.C>A missense	p.R61S novel	Ventricular septal defect	Heterozygous	Benign	Kassab et al., 2016 [35]
c.G>C missense	p.S19W	Atrial septal defect Tetralogy of Fallot	-	Possibly damaging	Kassab et al., 2016 [35]
c. 719G>A	P.G240D novel	Dilated cardiomyopathy	Heterozygous	Possibly damaging	Zhang et al., 2015 [45]
c.8A>G	p. Gln3Arg	Bicuspid aortic valve Aortic coarctation	-	Damaging	Bonachea et al., 2014 [51]
c.698T>C	p.L233P	Bicuspid aortic valve Aortic coarctation	Heterozygous Autosomal dominant	Damaging	Bonachea et al., 2014 [51]
c.46T>G	p. Y16D novel	Bicuspid aortic valve	Heterozygous	Disease causing	Shi et al., 2014 [52]
c.754A>C	p.T252P novel	Bicuspid aortic valve	Heterozygous	Disease causing	Shi et al., 2014 [52]
g.61051165A>G novel	NA	Ventricular septal defect	Heterozygous	-	Shan et al., 2014 [49]
g.61051463delC novel	NA	Ventricular septal defect	Heterozygous	-	Shan et al., 2014 [49]
g.61051227C>T novel	NA	Ventricular septal defect	Heterozygous	-	Shan et al., 2014 [49]
c.413A>T missense	p.Y138F	Atrial fibrillation	Heterozygous	-	Gu et al., 2012 [44]
c.628T>G missense	p.C210G	Atrial fibrillation	Heterozygous	-	Gu et al., 2012 [44]
c.394C>G	p.R132G novel	Atrial septal defect	Heterozygous	Disease causing	Jiang et al., 1998 [19]
c.569T>C	p.V190A novel	Ventricular septal defect Tetralogy of Fallot	Heterozygous	Disease causing	Jiang et al., 1998 [19]
c.667A>C	p.A266P novel	Tetralogy of Fallot	Heterozygous	Disease causing	Jiang et al., 1998 [19]
c.821A>G	p.H274R novel	Ventricular septal defect	Heterozygous	Disease causing	Jiang et al., 1998 [19]
c.56C>G	p.Ser19Trp	Bicuspid aortic valve	-	Possibly damaging	Padang et al., 2012 [47]
c.424T>C	p.Tyrl142His	Bicuspid aortic valve	-	Possibly damaging	Padang et al., 2012 [47]
c.551 G>T	pG184V novel	Atrial fibrillation	Heterozygous	Disease causing	Yang et al., 2012 [48]
c.653A>C	p.K218T novel	Atrial fibrillation	Heterozygous	Disease causing	Yang et al., 2012 [48]
c.796 G>C	p.A266P novel	Atrial fibrillation	Heterozygous	Disease causing	Yang et al., 2012 [48]
c.598T>G missense	p.W200G novel	Atrial fibrillation	Heterozygous	Disease causing	Wang et al., 2012 [15]
c.595C>G	p.L199 noel	Ventricular septal defect	Heterozygous	Pathogenic mutation Benign polymorphism	Wei et al., 2013 [34]

Pulmonary valve stenosis is a partial form of right ventricular outflow (RVOT) tract obstruction occurring due to the narrowing of the valve annulus causing stenosis [53]. It occurs alone in 8–10% of patients and is one of the most common forms of CHD [54]. Kassab et al., 2016, were the first to describe the direct association between biallelic *GATA5* alteration in humans and a complex phenotype of double outlet right ventricle, VSD, and PVS. Remarkably, out of 193 individuals with different forms of CHD, only one patient was found to have isolated pulmonary stenosis and be carrying a novel mutation of [chr20:g.61050383G>C (p.G63A)] located in the TAD that was inherited in an autosomal dominant pattern. However, there were no further details to ascertain the functional impact of this variant [35]. Regardless of the various *GATA5* variants identified to date, no patient has been reported with isolated PVS with this novel variant [Chr20:g.61041597C>G (p.Arg237Pro)].

### 4.2. GATA6 Variant and Complex CHD in Proband B

**Figure 5:**
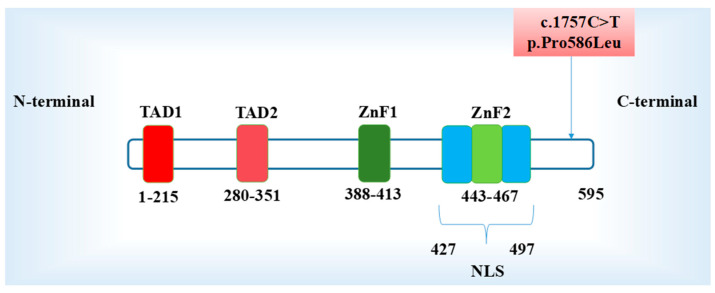
Schematic diagram of structural domains and their location on the GATA6 variant. The N-terminal consists of the two transcription activation domains (TAD1&2). A DNA-binding domain consisting of two similar zinc fingers (N-ZnF1, C-ZnF2) and the nuclear localization sequence (NLS). Upper light red box: Illustration of the GATA6 variant of interest in this study.

In this study, proband B presented with a complex CHD affecting the right-side structures resulting in early heart failure and requiring a heart transplant. A novel heterozygous [Chr18:c.1757C>T (p.Pro586Leu)] variant in the *GATA6* was identified. This missense variant is located on exon 7 resulting in the substitution of proline for a leucine at the C-terminal domain of GATA6 protein (Figure 5).

*GATA6* is located on human chromosome 18q11.1–18q11.2 and encodes a protein (GATA6) composed of 595 amino acids [23,37,55]. The amino acid composition of the GATA6 domains TAD1 and TAD2 are 1–215 and 280–351, respectively. The ZnF1 and ZnF2 domains have 388–413 and 443–467 amino acids, respectively, while the NLS domain has 427–497 amino acids [17]. The missense [Chr18: c.1757C>T (p. Pro586Leu)] variant substitutes proline for a leucine at the C-terminal domain of GATA6 protein (Figure 5).

The identified [Chr18:c.1757C>T (p.Pro586Leu)] variant is very rarely observed in the general population (minor allele frequency <0.1% in the ExAC database) and has not been previously reported in the literature in individuals with complex CHD. No established clinically significant variants identified could explain the primary clinical concerns in our patient. However, the inheritance of the variant from the affected father with TOF is consistent with an autosomal dominant pattern. Inheritance from an unaffected or mildly affected parent has also been reported in the literature [20]. The presence of modifier genes that impact cardiac phenotypes could cause incomplete penetrance and variable expressivity of CHD in some families [56]. Hence, GATA6 haploinsufficiency is most likely the cause of the proband’s complex clinical symptoms because it is predicted to interfere with crucial signaling molecules, inhibit valve reconfiguration, and affect the architecture of the extracellular matrix [57,58]. This highlights that haploinsufficiency or loss-of-function mutation in *GATA6* is possibly a mechanism underlying certain CHDs [2,59]. Our findings in ClinVar (Variation ID: 949027) revealed a missense mutation [(NM_005257.6):c.1757C>T: p.Pro586leu] that has not been detected in patients with GATA6-related disorders. The variant was characterized as a VUS because there was not enough information to clarify its involvement in causing disease. Therefore, this is the first report that supports the pathological impact of the mutant GATA6^p.Pro586leu^ protein.

GATA6 is expressed throughout the early stages of embryonic and fetal cardiac development in the myocardium, endocardium, neural crest, smooth muscle cells, and other cells generated from the secondary heart field (SHF) in the human heart [26]. It is essential for the maturation of SHF and the recruitment of cardiac neural crest cells, which together make up the cardiac outflow tract [60]. Importantly, the expression of GATA6 extends beyond that of GATA4 and GATA5 during cardiovascular development making it a critical contributor to heart formation and CHDs. Further, GATA6 is the only GATA member expressed in vascular smooth muscle cells (VSMCs) and plays a crucial role in regulating the phenotypes of these cells after vascular damage [61]. In humans, mutant *GATA6* zinc finger and NLS domains are unable to target the transactivation of genes involved in the semaphore-plexin pathway, which plays important roles in the development of the cardiovascular system [20,62]. When GATA6 is inactivated in the VSMCs and the cardiac neural crest cells, it results in fetal mortality due to conotruncal heart defects, further highlighting GATA6’s crucial role in regulating morphogenesis of the cardiac outflow tract and aortic arch [63].

Human variants of *GATA6* were first reported in individuals with persistent truncus arteriosus (PTA). However, over the years, pathogenic *GATA6* variants with autosomal dominant mode of inheritance have been reported in individuals with TOF, pulmonary atresia with ventricular septal defect (PA/VSD), PVS, DORV, and transposition of the great arteries (TGA) as well as isolated ASD, VSD, and atrioventricular septal defect (AVSD) [2,38,60].

Severe autosomal dominant CHDs such as complex right-sided anomalies including RV hypoplasia, TOF, and pulmonary valve atresia are also linked to missense variants in the *GATA6* [56]. Within affected families, pathogenic GATA6 variants are particularly prevalent. In addition, severely damaging nonsense or truncating frameshift variants in *GATA6* may result in biliary system abnormalities, congenital diaphragmatic hernia, pancreatic agenesis, and/or monogenetic diabetes [20,27,36,37,38,53,64,65].

As of 2019, over thirty *GATA6* mutations have been reported in human CHDs [26]. In recent times, 57 different types of GATA6 variants including exonic single-nucleotide variants, splicing errors, small insertions, and deletions have been described in moderate CHDs, and most of these mutations were found within the zinc finger and NLS domains, which are two of GATA6’s functional domains [66]. Our case is unique because it is the first case of this novel GATA6 variant in a family with a paternal history of CHD and an infant with severe CHD that affects the right-side structures and the ventricular septum leading to early heart failure requiring heart transplant.

### 4.3. Role of MicroRNAs in Targeting the GATA Family Genes in Heart Development

Lately, microRNAs’ (miRNAs’) posttranscriptional regulation has become a unique mechanism that is crucial to the regulation of embryonic development [67]. MicroRNAs (miRNAs) are small (18–25 nucleotides) non-coding RNA molecules that alter their target genes when they are expressed and are also involved in silencing and post-transcriptional regulation [68,69]. They either interfere with mRNA translation or disintegrate mRNA transcripts to control target genes [70]. They are currently being exploited as therapeutic targets for heart diseases and cardiomyocyte hypertrophy [68,69]. Different miRNAs have been identified to ensure appropriate differentiation, and proliferation in heart development and diseases [68,69]. Cardiomyocytes, endocardial cells, epicardial cells, and neural crest cells are the four main cell types that make up the mammalian embryonic heart throughout heart development [68,71].

In the last decade, several reports have shown the critical role of miRNAs in controlling cardiac transcription factors including GATA4, MEF2C, Tbx1, SRF or ISL1, and cardiac myosin heavy chain genes [67,68,72]. Certain miRNAs target particular genes, alter transcription factors, and control the growth of cardiomyocytes [67,68,72]. For instance, studies have shown that miR-200c directly targets the GATA4. In human embryonic stem cells, GATA4 protein levels increase when miR-200c is knocked down and decrease when miR-200c is overexpressed, thereby demonstrating the crucial role of miRNA control of a GAT4 gene in the development of CHD [70]. Another transcription factor that is a novel target of miR-218 is the RE1-silencing transcription factor (REST). REST controls the Wnt/β-catenin signaling pathway and GATA4 to control the cardiac development of embryonic stem cells. This mechanism further provides evidence of the crucial role of miRNA in cardiomyocyte hypertrophy targeting GATA4 [68,72].

## 5. Conclusions

To the best of our knowledge, this is the first report of two novel heterozygous *GATA5* and *GATA6* variants, p.Arg237Pro and p.Pro586Leu, identified in two patients with severe pulmonary valve stenosis and complex right-sided CHDs, respectively. Consistent with the dominant pattern of pathogenic variants in the members of GATA family genes, the severe and complex clinical features of proband A and B highlight that the expression of only one healthy allele of either *GATA5 or GATA6* is likely insufficient to perform the biological function of the protein that is required for normal cardiac formation. Even though we did not perform further analysis to determine the functional effect of the changes in the mutant variants, our report provides valuable insight into the variable penetrance and expressivity, and the critical impact of GATA5 and GATA6 perturbations in complex right-sided cardiac defects as well as the strength of WES in identifying novel genetic variations. Importantly, proband A was first diagnosed at day 5 of life through postnatal CHD screening, indicating the specificity and sensitivity of employing this routine screening modality in detecting critical CHDs for all newborn infants [73]. Furthermore, proband B was first diagnosed with CHD prenatally, yet it did not prevent postnatal complications. Hence, the discovery of this novel variant in the family will serve as the basis for well-informed decision-making and the option for assisted reproductive techniques in subsequent pregnancy. Additionally, deeper insight into miRNAs’ role during heart development may help identify potential treatment targets for congenital heart diseases, and heart failure and also pave the way for personalized treatment options in affected individuals.

## Figures and Tables

**Table 1:** *GATA1/2/3* gene-related human disorders that have been described.

*Gene*	*GATA1*	*GATA2*	*GATA3*
Disorders	X-linked dyserthropoietic anemia, thrombocytopenia, X-linked Diamond-Blackfan (DBA) anemia, transient myeloproliferative disorder (TMD), Down syndrome acute megakaryoblastic leukemia (DS-AMKL)	Monocytopenia with mycobacteria infections (MonoMac), Dendritic cell, B, and natural killer(NK) lymphoid deficiency, Familial Myelodysplastic syndromes/Acute Myeloid Leukemia (MDS/AML), Emberger syndrome (primary lymphedema with MDS)	HDR syndrome (hypoparathyroidism, deafness, and renal dysplasia)

**Table 2:** Cardiac defects linked with GATA4/5/6 (modified from Whitcomb et al., 2020) [26].

Cardiac Phenotypes	GATA4	GATA5	GATA6
Atrial fibrillation	√	√	√
Aortic stenosis	√	√	
Atrial septal defects	√	√	√
Atrio ventricular septal defects	√	√	√
Bicuspid aortic valves	√	√	√
Dilated cardiomyopathy	√	√	√
Double inlet left ventricle	√		
Double outlet left ventricle			√
Double outlet right ventricle	√	√	√
Dextro-transposition of the great arteries		√	
Hypertrophic cardiomyopathy	√	√	
Major aortopulmonary collateral arteries			√
Patent ductus arteriosus	√	√	√
Pulmonary stenosis	√	√	√
Persistent ductus arteriosus	√	√	√
Pulmonary atresia with ventricular septal defects			√
Truncus arteriosus	√		√
Transposition of the great arteries			√
Tetralogy of Fallot	√	√	√
Ventricular septal defect	√	√	√

**Table 3:** Proband A’s *GATA5* quad whole-exome sequencing.

Gene	Genomic Change	Nucleotide Change	Transcript	Protein Change	Classification	Zygosity	Inheritance
*GATA5*	Chr20:g.61041597C>G	c.710G>C	NM_080473.4 GRCh37/hg19	p.Arg237Pro	VUS	Heterozygous	Mother (not in maternal uncle)

**Table 4:** Proband B’s *GATA6* Trio exome sequencing.

Gene	Genomic Change	Nucleotide Change	Transcript	Protein Change	Classification	Zygosity	Inheritance	Minor Allele Frequency
*GATA6*	Chr18:g.19780755	c.1757C>T	NM_005257.5 GRCh37/hg19	p.Pro586Leu	VUS	Heterozygous	Father	<0.1%

## Data Availability

The datasets used and analyzed during the current study are available from the corresponding author upon reasonable request. The study was registered in dbGaP under Novel Gene-Environment Regulatory Circuit in Chamber-Specific Growth of Perinatal Heart, Study ID: 45333. The stable dbGaP accession for this study is phs002725.v1. p1.

## Abbreviations

ACMG	American College of Medical Genetics and Genomics
AMP	Association for Molecular Pathology
ASD	Atrial septal defect
CHD	Congenital heart defect
DS	Down syndrome
EKG	Electrocardiogram
GATA 5	GATA-binding factor 5
GATA 6	GATA-binding factor 6
IRB	Institutional Review Board
MPA	Main pulmonary artery
NICU	Neonatal intensive care unit
NLS	Nuclear localization sequence
NSVD	Normal spontaneous vaginal delivery
PA	Pulmonary atresia
PBMCs	Peripheral blood monocytes
PTA	Persistent truncus arteriosus
PVS	Pulmonary valve stenosis
REST	RE1-silencing transcription factor
RVH	Right ventricular hypoplasia
SHF	Secondary heart field
SNP	Single-nucleotide polymorphism
TAD	Transcription activation domain
TF	Transcription factor
TGA	Transposition of great arteries
TOF	Tetralogy of Fallot
UCLA	University of California, Los Angeles
VSD	Ventricular septal defect
VSMCs	Vascular smooth muscle cells
VUS	Variant of uncertain clinical significance
WES	Whole-exome sequencing
ZnF	Zinc finger
